# The Convalescent Home at Yarmouth

**Published:** 1888-03-24

**Authors:** 


### THE CONVALESCENT HOME AT YARMOUTH.

Miss Lucy Hansell would feel obliged to the Editor of The Hospital if he would correct an error made by "Our Roving Correspondent" in his account of the Children's Convalescent Home at Great Yarmouth. He says, in the number for February 18, that the home is closed during March and April. This is not the case. There are now sixteen children in the home, and doing well and growing strong, notwithstanding the bitterly cold weather there has been. Last year the home was closed in March and April because of the alterations—making two houses into one; so no doubt this led to the mistake.

Upper Close, Norwich.

[London children are sent to the Children's Convalescent Home at Yarmouth, and Miss Lucy Hansell thinks if it is seen in The Hospital that the home is closed during March and April it may prevent their being sent.]

